# What Are the Factors That Can Challenge and Promote Resilience in Parents of Children With Intellectual Disabilities? A Systematic Review

**DOI:** 10.1111/jar.70165

**Published:** 2025-12-25

**Authors:** Wattana Tejakum, Maria Truesdale, Craig Melville, Deborah Cairns

**Affiliations:** ^1^ School of Health and Wellbeing, College of Medical, Veterinary & Life Science University of Glasgow Glasgow UK; ^2^ Boromarajonani College of Nursing Bangkok, Faculty of Nursing Praboromarajchanok Institute Bangkok Thailand

**Keywords:** children, intellectual disabilities, parents, resilience

## Abstract

**Background:**

Raising a child with intellectual disabilities poses significant challenges. Despite the challenges faced by parents of children with intellectual disabilities, resilient parents can maintain a positive outlook and effectively navigate these challenges, whereas others struggle. This systematic review contributes to the literature by synthesising research on the factors influencing resilience among parents of children with intellectual disabilities.

**Methods:**

Searches were conducted using databases MEDLINE, PsycINFO, EMBASE, CINAHL and SocINDEX. Reference lists of all included articles were searched. Quality assessments were performed using CASP.

**Results:**

Of the 6382 studies identified, 27 were included in the review. A narrative synthesis of the included studies was performed. The study details were organised into two main factors: personal and contextual.

**Conclusion:**

This systematic review highlights the need to promote the resilience of parents of children with intellectual disabilities. Evidence suggests that a combination of personal and contextual factors contributes to parental resilience.

## Lay Summary

- This systematic review identified crucial factors associated with resilience in parents of children with intellectual disabilities across 12 countries.
- The insights gained emphasise the importance of tailored interventions and services to enhance resilience in these parents.
- This review highlighted the influence of gender on parents' perceptions of resilience, calling for a nuanced understanding of resilience among caregivers.
- This systematic review acknowledges the dynamic nature of resilience across different caregiving phases; however, it highlights a gap in the analysis by not providing a detailed breakdown of the caregiving trajectories. This absence limits the understanding of the unique challenges at different points, emphasising the importance of future research in this domain.

## 1 Background

Parenting is an intricate journey that takes on a unique and often challenging dimension when a child is diagnosed with intellectual disabilities (Rydzewska et al. 2021). Resilience in parents of children with intellectual disabilities is crucial for their ability to cope, adapt, and provide optimal support to their children (Halstead et al. 2018). While existing reviews (Ilias et al. 2018; Iacob et al. 2020) have examined resilience in parents of children with autism and developmental disabilities, they do not focus exclusively on parents of children with intellectual disabilities.

Caring for a child with an intellectual disability poses distinct challenges that differ from those associated with other developmental disorders. Intellectual disability is defined by substantial limitations in intellectual functioning and adaptive behaviour, which impact the ability to learn, communicate, and perform daily activities (APA 2013). These profound deficits lead to long‐term and often intensive caregiving demands. This systematic review aims to fill this gap by concentrating specifically on this population and offering a more targeted exploration of resilience factors.

Parents are required to assist with basic daily tasks such as hygiene, feeding, and mobility (Wu and Boat 2015; Marrus and Hall 2017), while also managing their child's behavioural challenges, including aggression and self‐injury, and the delay of developmental milestones (Baker et al. 2003; Harris 2010). In addition, they are often confronted with societal prejudice and stigma (Mitter et al. 2019; Tejakum et al. 2022), which further compounds their emotional burden.

Mental health problems such as anxiety, depression, and stress are more prevalent in mothers of children with intellectual disabilities than in those raising typically developing children (Rydzewska et al. 2021). The cumulative stress of long‐term caregiving has been linked to reduced psychological well‐being, poorer maternal physical health, and increased parenting stress (Lee 2013; Crnic et al. 2017; Gogoi et al. 2017).

Despite these challenges, some parents demonstrate a capacity to adapt, sustain their well‐being, and foster positive family functioning. Supportive co‐parenting relationships have been found to reduce parenting stress and decrease child behavioural issues (Zhu et al. 2024). Access to social support, including emotional, informational, or practical support, has also been identified as a key factor in enhancing the quality of life for families of children with intellectual disabilities (Hassanein et al. 2021). Parents who successfully navigate these demands often facilitate better developmental outcomes for their children and greater family resilience (Widyawati et al. 2023). Moreover, interventions focusing on family support, social skills and emotional socialisation can help parents manage stress and promote positive adaptation (Jacobs et al. 2019).

Understanding what empowers parents to manage heightened mental health challenges is essential, with resilience being a potential explanation. Contemporary understandings of resilience emphasise its nature as a dynamic process of adaptation in the face of adversity rather than a fixed trait (Luthar and Cicchetti 2000; Masten 2018). For example, Rutter (1987) conceptualised resilience as an individual's ability to overcome adversity despite exposure to risk factors. Luthar and Cicchetti (2000) further defined it as “a process wherein individuals display positive adaptation despite experiences of significant adversity”. Walsh (2015) described resilience as the ability to withstand hardship and emerge stronger, whereas Masten (2018) framed it as the capacity of a system, individual, family, or community to adapt successfully under stress. These perspectives collectively position resilience as an adaptive, multifaceted, and relational process.

In line with this conceptualization, the current review treats outcomes such as sustained mental health, effective family functioning, and positive psychological adaptation as valid resilience indicators. Such outcomes not only reflect successful coping but also signal the presence of resilient mechanisms. Therefore, identifying the factors that support these outcomes is essential for informing the development of effective, evidence‐based interventions for families of children with intellectual disabilities.

Given the significant mental health risks faced by these parents, it is critical to understand what enables some of them to thrive despite adversity. The observation that some parents achieve “positive adaptation outcomes” despite these risks raises a vital question: what factors are associated with resilience in this population? To address this question, the present systematic review synthesises current evidence on resilience in parents of children with intellectual disabilities, with the aim of synthesising evidence on both the stressors that challenge resilience (low levels of stress might indicate higher levels of resilience) and the personal and contextual factors (resources, protective factors) that promote it. Additionally, the review explores gaps in the literature, including differences in resilience between mothers and fathers and variations across different caregiving stages. The specific research questions were as follows:

1. What are the factors that can challenge and promote resilience in parents of children with intellectual disabilities?
2. Are there evidence gaps in resilience research regarding the differences between mothers and fathers of children with intellectual disabilities and variations in different caregiving stages?

## 2 Method

**FIGURE 1:**
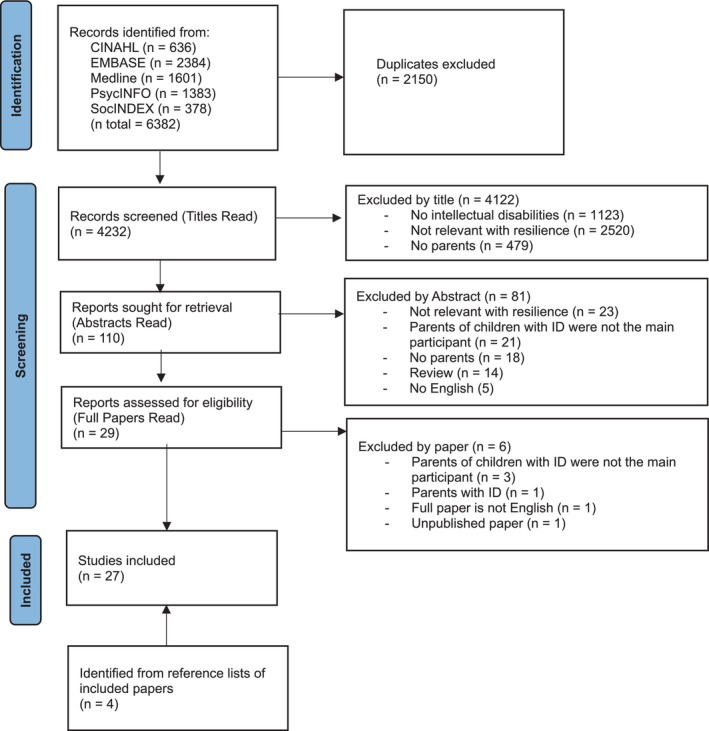
PRISMA (Shamseer et al. 2015).

This systematic review was registered with the International Prospective Register of Systematic Reviews (PROSPERO) (registration number: CRD42022335724) and followed the Preferred Reporting Items for Systematic Reviews and Meta‐Analyses (PRISMA) guidelines, flow diagram and checklist (Figure 1) (Shamseer et al. 2015). A systematic search was conducted using five databases: MEDLINE, PsycINFO, EMBASE, CINAHL and SocINDEX from June 2022 to June 2023. Key search terms included mental retardation, intellectual disabilities, learning disabilities, developmental disabilities, parents, resilience, coping, adaptation, hardiness, combined using Boolean operators (AND, OR) and truncation (*) (Appendix A). The search was limited to English‐language studies with no time restrictions. We excluded the term “stress” to focus on the outcome as the concept of resilience, as including stress might introduce variables that are less relevant to our topic. The inclusion and exclusion criteria were as follows:

### 2.1 Study Selection

*Inclusion criteria*:

1. Parent who has children with intellectual disabilities.
2. Studies focusing on the concept of resilience.
3. Empirical study, cross‐sectional studies, cohort studies, case–control study and qualitative study.
4. Studies from peer reviewed journals.
5. Full‐text papers written in English.

*Exclusion criteria*:

1. Parents with a diagnosis of intellectual disabilities.
2. Studies focusing solely on stress without an explicit link to resilience.
3. Information about parents of children with intellectual disabilities was not separately presented and/or parents of children with intellectual disabilities were less than 80% of the total sample.
4. Parents of children with other disabilities (autism, a specific learning difficulty in reading or writing, physical disability).
5. Review papers, theoretical articles, commentaries, and unpublished papers.

The study selection process involved the initial screening of papers by title and abstract by the first reviewer (W.T.). The second (M.T.) and third (D.C.) reviewers checked 10% of the sample randomly. W.T. is a doctoral fellow, M.T. a senior lecturer and D.C. a professor. Disagreements between the two studies were resolved through consensus discussions. These two studies were ultimately excluded because they did not meet the inclusion criteria, which required that the parents of children with intellectual disabilities be the main participants. Three reviewers assessed the full texts for eligibility. Data extraction was performed in a structured format (Table 1). The researchers also reviewed the reference lists of the included studies to ensure comprehensive coverage.

**TABLE 1:** Included studies.

	Author (year), country	Participants	Intellectual disability level	Objective	Methodology	Measures	Results	Theory	Apprasal (CASP score)
1	Azar and Badr (2006), Lebanon	127 mothers aged 25–56 years Children with intellectual disabilities aged 2–18 years	Mild: 47 Moderate: 61 Severe: 16 Profound: 3	To identify factors that influence the adaptation of mother of children with intellectual disabilities	Quantitative study, a cross‐sectional design	1. The Family Inventory of Life Events and Changes (FILE) 2. The Parenting Stress Index (PSI) 3. The Family Crisis Oriented Personal Evaluation Scale (FCOPES) 4. The Centre of Epidemiologic Studies‐Depression Scale (CES‐D)	1. Families with high income and medical insurance have less strain than low‐income families with no insurance (*p* = 0.00, *p* = 0.01) 2. Family income related with depressive symptoms 3. Mothers with less education have less coping resources (*p* = 0.04) 4. Family strain was predictor of depressive symptoms (*p* = 0.00), maternal stress the second (*p* = 0.00), followed by family income (*p* = 0.03)	—	B1
2	Bayrakli and Kaner (2012), Turkey	491 mothers aged 23–63 years (257 mother of children with intellectual disabilities and 234 mothers of children with typical development) Children with intellectual disabilities aged 4–26 years Children with typical development aged 5–15 years	N/A	To investigate the relationship between resiliency perceptions, perceived quantity and quality of social support and perceived coping strategies of the mothers	Quantitative study, a cross‐sectional design	1. Mother Resilience Scale (MRS) 2. Revised Parental Social Support Scale (RPSSS) 3. Coping Style Scale (CSS)	1. The quantity of social support has a positive effect on problem‐focused coping (*p* < 0.05) and resiliency (*p* < 0.05) 2. Problem‐focused coping affects resiliency positively (*p* < 0.05) 3. The quality of social support has a positive effect on problem‐focused coping (*p* < 0.05) and resiliency (*p* < 0.05).	—	A
3	Ben‐Zur et al. (2005), Israel	100 mothers aged 41–85 years Children with ID aged 20–56 years	N/A	To examine the mental health, resources and stress among mothers who keep their adult child at home vs. those who choose placement in a community arrangement.	Quantitative study, a cross‐sectional design.	1. Demographic Questionnaire 2. Mental Health Inventory (MHI) 3. Questionnaire on Resources and Stress (QRS‐F) 4. Interpersonal Support Evaluation List (ISEL) 5. Hardiness	1. The mental health score correlated positively with both social support and hardiness (*p* < 0.001) 2. Stress related negatively to mental health, social support and hardiness (*p* < 0.001) 3. Mothers’ economic situation, years of education and perceived health correlated positively with mental health, social support and hardiness, and related negatively to stress (*p* < 0.001)	—	B1
4	Caples et al. (2018), Ireland	79 mothers and 16 fathers, aged 28–57 years Children with Down syndrome aged 0–30 years	N/A	To examine links between family demands, family appraisal, family resources, family problem‐solving communication and family adaptation	Quantitative study, a cross‐sectional design	1. Demographic questionnaire 2. The Family Index of Regenerativity and Adaption General 3. The Family Management 4. The Family Problem Solving Communication 5. The Family Member Well‐Being 6. The Brief Family Assessment	1. Family resources (family hardiness, relative and friend support) and family problem‐solving (affirmative communication) are the positive predictors of family functioning (*p* < 0.05)	Resiliency Model of Family Stress, Adjustment and Adaptation (McCubbin et al. 1996)	B1
5	Choi and Yoo (2015), Korea	126 parents (117 mothers and 8 fathers) aged 25 to > 40 years Children with Down syndrome aged 0–15 years	N/A	To identify the factors related to resilience of the families of children with Down syndrome	Quantitative study, a cross‐sectional design	1. DS‐related health 2. Resource &Stress 3. Depression 4. Strain and stress 5. Stigma 6. Emotionality, Activity, Sociability 7. Family Adaptability & Cohesion 8. Family Attachment and Changeability 9. Family Problem Solving and Communication 10. Self‐perceived 11. Family Coping 12. Healthcare 13. Community services 14. The Family APGAR	1. Family adaptation was negatively related to child age (*p* < 0.001), developmental level of child (*p* = 0.023), parent depression (*p* < 0.001) and strain and stress (*p* < 0.001). 2. Family adaptation was positively related to health of parents (*p* = 0.011), communication skills (*p* < 0.001), cohesiveness (*p* < 0.001), flexibility (*p* < 0.001), supportive friends/relatives (*p* < 0.001) and available community services (*p* = 0.004).	Family resilience Patterson's model (Patterson,2004)	B2
6	Cless et al. (2018), USA	351 mothers aged 16–70 years Children with Down syndrome mean aged 7.67 years	N/A	To understand mothers’ experiences of children with Down syndrome, in understanding relationship among coping, hope, and relationship quality	Quantitative study, a cross‐sectional design	1. The Family Crisis Oriented Personal Evaluation Scales 2. The Herth Hope Index 3. Relationship quality 3.1 The RDAS 3.2 The CSI	1. Hope was significantly correlated with religious coping (*p* < 0.001), internal coping (*p* < 0.001), seeking support coping (*p* < 0.001) 2. Hope was significantly correlated with dyadic adjustment (*p* < 0.001), relationship satisfaction (*p* < 0.001) 3. Greater religious coping was significantly associated with more hope (*p* < 0.001) 4. Greater internal coping was significantly associated with more hope (*p* < 0.001)	The Contextual Model of Family Stress (Boss et al. 2016) which based on the original ABCX model (Hill 1949)	B1
7	Costigan et al. (1997), USA	165 families (father 121, mothers 162) mean aged 36 years Children with mental retardation aged 6–18 years A comparison 52 families (fathers 36, mothers 50) mean age 40 years Children with typical development aged 6–18 years	Mild: 110 Moderate: 55	To evaluate how the presence of children with mental retardation in the family influenced family problem‐solving interactions on family adaptation, which emphasise either family disruption or family resilience	Quantitative study, a longitudinal study	A series of family the administration of questionnaire	1. The single mothers in the MR group (*M* = −0.10, SD = 0.67) displayed less supportive problem solving than the single mothers in the comparison group (*M* = 0.83, SD = 1.14) 2. The mothers engaged in relatively more supportive problem solving (*M* = 0.14, SD = 0.76) than the fathers (*M* = −0.20, SD = 0.64) 3. The mothers displayed more active listening (*M* = 0.04, SD=.67) than the fathers (*M* = −0.15, SD = 0.76)	The double ABCX model of adjustment and adaptation (McCubbin and Patterson 1983)	C1,4
8	Dukmak (2009), United Arab Emirates (UAE)	63 parents (41 mothers and 22 fathers, 47% of parents aged below 40 years) Children with intellectual disabilities, also had a diagnosis of either Down syndrome, Cerebral palsy, or Autism aged 6–10 years	Moderate and Severe (The study not presented of how many children of each level)	To examine the relationship between child with intellectual disabilities and parent characteristics and family adaptation, ways of coping, and parenting satisfaction	Quantitative study, a cross‐sectional design	1. Questionnaire on Resources and Stress Scale—Short Form to assess a family's adaptation 2. Ways of Coping Scale 3. Parenting Satisfaction Scale	1. Emotional‐focused coping correlated with family adaptation (*p* = 0.0001) 2. Problem‐focused coping correlated with parenting satisfaction (*p* < 0.001)	—	B2
9	Ellingsen et al. (2014), USA	100 mothers of children with developmental delay 132 mothers of children with typical development mean age 32–34 years Children aged 3–5 years	Borderline or mild or moderate (the study not presented of how many children of each level)	To examined factors that promoted effective parenting (resilient) in the presence of child developmental delay, high child behaviour problems, and low family income	Quantitative study, a longitudinal design	1. Bayley Scales of Infant Development 2. Child Behaviour Checklist 3. Family Information 4. Life orientation Test‐Revised 5. Parent–Child Interaction Rating Scale	1. Education and optimism appeared to be protective factors for positive parenting at age 3 and 5 (*p* < 0.05) 2.Health appeared to be an additional protective at age 5 (*p* < 0.05) 3. Low income, child's development negatively effect with positive parenting (*p* < 0.05)	ABCX model (McCubbin et al. 1996)	A
10	Gardner and Harmon (2002), Australia	6 mothers aged N/A Children with intellectual disabilities aged 1–18 years	N/A	To explore resilience from a parent's perspective	Qualitative study	Interview open‐ended questions around the issue of caring for a child with intellectual disabilities from six resilient mothers	Key themes: 1. Developing a sense of self 2. The emotional journey 3. Being a team 4. The power to act 5. Being organised 6. Using supports 7. My cup's half full 8. I also have needs 9. No one misses out 10. Making sense of life	—	B3
11	Gerstein et al. (2009), USA	115 families (the study did not report on a number of mothers or fathers) aged N/A Children with intellectual disabilities aged 3–5 years	Developmental level was determined by using the BSID‐II (the study not presented on how many children have each level)	‐ To examine the trajectories of daily parenting stress in families of children with intellectual disabilities ‐ To explore risk and factors that lead to more resilient parent	Quantitative study, a longitudinal design	1. Developmental level the BSID‐II 2. Parenting daily hassles 3. Well‐being Symptom Checklist 4. Dyadic Adjustment 5. Parent–Child Interaction	1. Mothers reported significantly higher levels of growth models of parenting daily hassles (PDH) than fathers at 48 and 60 months (*p* < 0.001) 2. Decreases in mothers’ daily parenting stress trajectory were associated with both mother and father's well‐being (*p* < 0.05) and perceived marital adjustment, as well as a positive father–child relationship (*p* < 0.05)	—	B2
12	Ginevra et al. (2018), Italy	152 parents (62 fathers and 90 mothers) mean aged 51.25 years Children with intellectual disabilities aged 10–22 years	Mild: 147	To analyse the role of career adaptability and resilience on parents of children with mild intellectual disabilities’ life satisfaction	Quantitative study, a cross‐sectional design	1. Career Adapt‐Abilities Scale 2. The Connor‐Davidson Resilience Scale (CD‐RISC) 3. The Satisfaction with Life Scale	Career adaptability (*B* = 0.36, 95% CI [0.152, 0.579]) had a significant indirect link with life satisfaction through the mediating role of resilience	—	B1
13	Grant and Whittell (2000), UK, Wales	27 families (15 fathers, 25 mothers, 1 sister and 1 cousin) aged N/A Children with intellectual disabilities aged 0 to more than 50 years	N/A	To understand the differentiated nature of coping regard to gender, life stage and family composition	Qualitative study	In‐depth interviewing	Key themes: 1. Mothers are more effective coping skills than fathers 2. Couples have adopted a ‘sharing‐caring’ strategy 3. Unaccompanied caregivers emphasised cognitive coping 4. Parents with pre‐school children have less confidence in knowledge, skill, and capacity to handle difficult situations 5. Caregivers of school‐aged and younger adults emphasised problem‐solving techniques 6. Elder caregivers were more resigned to their role and shown acceptance of the current situation	Transactional model of coping	B1
14	Hsiao and Van Riper (2011), Taiwan	83 families (75 fathers and 80 mothers) mean aged 38.68 years Children with intellectual disabilities aged 4–17 years	N/A	To examine the effects of family demographics, family demands, and family appraisal on adaptation in Taiwanese families of children with Down syndrome.	Quantitative study, a cross‐sectional design	1. Family Strains Index 2. Family Stressors Index 3. Family Management Measures 4. Chinese Health Questionnaire 5. Family Assessment Device	1. Gender (*p* < 0.01), family demands (*p* < 0.001), and family appraisal (*p* < 0.001) associated with health 2. Age of the child (*p* < 0.01), family demands (*p* < 0.001), and family appraisal (*p* = 0.001) significantly associated with family functioning 3. Family appraisal partially mediated the relationship between family demands and individual and family adaptation (*p* < 0.001)	The Resiliency Model of Family Stress, Adjustment, and Adaptation (McCubbin et al. 1996)	B2
15	Hsiao (2014), Taiwan	155 parents (80 mothers and 75 fathers) aged N/A Children with Down syndrome aged < 18 years	N/A	To examine how family demographics, family demands and social support related to family functioning	Quantitative study, a cross‐sectional, design	1. Family demographic information 2. Family demands: Family Stressors Index, Family Strains Index, Family Management Measure 3. Chinese version of the Perceived Social Support Scale 4. The Chinese version of the General Functioning Scale	1. Families with older age of children (*p* < 0.001), greater parental education (*p* < 0.01), higher family income (*p* < 0.001), less family demands (*p* < 0.001) and greater social support (*p* < 0.001) experienced healthier family functioning 2. Family demands significantly predicted social support (*p* < 0.001) 3. Family demands significantly predicted family functioning (*p* < 0.001) 4. Social support significantly predicted family functioning (*p* < 0.001)	—	B2
16	John and Zapata Roblyer (2017), India	47 mothers aged N/A Children with intellectual disabilities (*n* = 21), and an associated diagnosis of Down syndrome (*n* = 2), Autism (*n* = 9), Cerebral palsy (*n* = 10), Epilepsy (*n* = 2), and syndrome disorders (*n* = 3) aged 3–6 years	N/A	To examined relevance of the key constructs of the stress and resilience framework in the urban Indian context	Qualitative study	Interview questions: Reaction to Diagnosis Interview	Key themes: *Appraisal of the situation* 1. Negative appraisals 2. Pragmatic and benefit 3. Evolving appraisals 4. Appraisals of cause of disability *Stressors* 1. Diagnosis 2. Demands on time and money 3. Marital and family conflicts 4. Child's behaviour, health, and functioning 5. Reproductive choices *Resources* 1. Personal resources 2. Family‐level resources 3. Societal resources	The Double ABCX Model (McCubbin and Patterson 1983)	B1
17	Karaca and Konuk (2021), Turkey	28 mothers mean age 36.9 years Children with developmental disabilities (also had a diagnosis of either Down syndrome, intellectual disabilities) aged 8–17 years	The IQ levels 20–35 (the study not presented on how many children have each level)	To explore the spiritual needs of mothers of children with developmental disabilities and analysed the effect of spirituality on their lives.	Qualitative study	Interview questions: a semi‐structured form	Key themes: 1. The journey to acceptance 2. The meaning of life 3. Concerns the future 4. Coping strategies	—	B1
18	Lloyd and Hastings (2009), England	138 mothers and 58 fathers aged 23–57 years Children with intellectual disabilities (*n*=40), Down syndrome (*n* = 26), Cerebral palsy (*n* = 16), and Autism (*n* = 56) aged 3–18 years	N/A	To explore hope in parents of children with intellectual disabilities consistent with the main psychological theory of hope	Quantitative study, a cross‐sectional design	1.Positive Affect scale of the Positive and Negative Affect Schedule 2.The Parent and Family Problems 3.Hospital Anxiety and Depression Scale	1. Mothers: lower levels of hope (*p* = 0.000) and more child behaviour problems (*p* = 0.000) predicted maternal depression (*p* = 0.000), positive affect was predicted by less problematic child behaviour (*p* = 0.019) and by higher levels of hope (*p* = 0.000) 2. Fathers: Anxiety and depression were predicted by low hope (*p* = 0.037), (*p* = 0.000), positive affect was predicted by high hope agency (*p* = 0.000).	Snyder's hope theory	B2
19	Lustig and Thomas (1997), USA	117 parents (66 mothers mean age 51.8 and 51 fathers mean age 53.1) Children with mental retardation aged 18‐30 years	N/A	To evaluate family adaptation during when an adult child with mental retardation is adjusting to initial entry into supported employment	Quantitative study, a cross‐sectional design	1. The Family Coherence 2. Family Hardiness 3. Family Bonding 4. Family Flexibility 5. Supported Employment Family Concerns 6. Family Member Wellbeing	Regression analysis indicated a significant relationship between the score on the four family strength measures and family adaptation (*p* < 0.05)	Resiliency Model of Family stress, Adjustment and Adaptation	B1
20	Mohan and Kulkarni (2018), India	32 parents (29 mothers and 3 fathers) aged 21–70 Children with intellectual disabilities aged 6–28 years	N/A	To identify resilient response patterns among parents of children with intellectual disabilities	Qualitative study	Interview the dominant themes with respect to resilience that emerged were acceptance, cognitive adaptation, positive affect, social support and self‐efficacy	Key themes: 1. Individual factors – Perception – Cognition – Emotion – Behaviour 2. Child‐related factors – Physical appearance – Age of the child 3. Contextual factors – Social support – Socioeconomic status	—	B2
21	Poehlmann et al. (2005), USA	10 families of children with Down syndrome 11 families of children with fragile X syndrome Children aged 11–23 years	N/A	To examine the mothers’ experiences surrounding their children's diagnosis, including positive experiences and potential challenges	Qualitative study	Interview with open‐ended questions and semi‐structured interview	Key themes: 1. Perception of a child's disability 2. Seeking social support from siblings, family members, church, friends were instrumental in their initial and ongoing adaptation 3. Relying on faith or religion 4. Emotional‐focused coping strategies	—	B2
22	Rajan et al. (2016), India	60 parents (30 mothers, 30 fathers) aged 26–60 years Children with intellectual disabilities aged 5–22 years	Mild: 30 Moderate: 30	To examine the resilience across the parent and child related demographic factors	Quantitative study, a cross‐sectional design	Connor Davidson Resilience Scale (CD‐RISC)	1. Parental education made a significant difference in their resilience (p < 0.01) 2. The study did not elicit a statistically significant relationship of resilience with parental age, years lived with child after diagnosis, child's age, gender and IQ	—	B2
23	Rajan and John (2017), India	121 parents (111 mothers, 6 fathers, 4 grandmothers) aged 21–60 Children with intellectual disabilities aged 1–30 years	Mild: (46%) Moderate: (31%) Severe: (18%) Profound: (5%)	To explore the resilience of parents and its relationship with the impact of child's disability	Quantitative study, a cross‐sectional design	1. Connor Davidson Resilience Scale (CD‐RISC) 2. NIMH Disability Impact Scale (NIMH‐DIS)	1. Parents experienced lower resilience when they evaluated the impact of child's disability more negatively (*p* < 0.01) 2. Parental positive perceptions of the disability associated with the experience of greater resilience (*p* < 0.01)	—	B1
24	Rajan et al. (2018), India	57 parents (28 mothers, 29 fathers) aged 26–61 years Children with intellectual disabilities aged 5–22 years	Mild: 27 Moderate: 30	To examine the variations in parental resilience with their locus of control orientations	Quantitative study, a cross‐sectional design	1. Connor‐Davidson Resilience Scale (CD‐RISC) 2. Internal‐External Scale	1. Parents having with internal locus of control (*M* = 71.38, SD = 12.79) experienced greater resilience when compared to external locus of control (*M* = 51.08, SD = 13.41), (*t* = 4.84, df = 55, *p* < 0.001)	Theoretical model of resilience (Olsson 2008)	B3
25	Raspa et al. (2014), USA	1099 families (mothers 89%) mean age 46.8 years Child with Fragile X syndrome (FXS) aged 1–65 years	N/A	To examine factors in family adaptation to FXS	Quantitative study, a cross‐sectional design	1. Parenting knowledge 2. Social support 3. Respondent well‐being 4. Family social life 5. Impact and quality of life	1. Social supports were interrelated (*p* < 0.001) to parenting knowledge, well‐being and quality of life which are the positive adaptation 2. Financial impact negatively related (*p* < 0.001) with well‐being, social life, family impact and quality of life	—	B2
26	Truitt et al. (2012), USA	446 caregivers aged 21–84 years Children with Down syndrome aged 0–48 years	N/A	To examine the relationship of uncertainty and hope with adaptation in caregivers of children with Down syndrome	Quantitative study, a cross‐sectional, correlational research design	1. The Parental Perceived Uncertainty Scale 2. The Trait Hope Scale 3. The 20 items scale adaptation 4. Goals for child (focus of hope)	1. Hope for self was found to be positively associated with adaptation (*p* < 0.01) 2. Uncertainty was negatively associated with both hope for self (*p* < 0.01) and adaptation (*p* < 0.01)	Uncertainty, hope, and adaptation	B1
27	Van Riper (2007), USA	76 mothers aged N/A Children with Down syndrome aged 0–15 years	N/A	To examine linkages between family demands, family resources, family problem solving and coping, and family adaptation in families of children with Down syndrome	Quantitative study, a cross‐sectional design	1. Demographic information 2. Individual adaptation 3. Family adaptation 4. The Family Inventory of Life Events 5. The Family Inventory of Resources for Management 6. The Family Problem‐Solving Communication 7. The Family‐Crisis‐Oriented Personal Evaluation Scales	1. Family demands were significantly associated with family adaptation (*p* < 0.01) 2. Family resources (Family strengths, health, family support) were significantly associated with family adaptation (*p* < 0.01) 3. Problem‐solving communication was significantly associated with family adaptation (*p* < 0.01)	Resiliency Model of Family Stress, Adjustment, and Adaption (McCubbin and MaCubbin 1993)	B2

### 2.2 Quality Assessment

The quality of the studies was assessed using the Oxford Critical Appraisal Skills Program checklist (CASP 2018). This included the clarity of the research objectives, methodology, study design and size, participant selection criteria, measures used, data collection methods, analytical approaches, findings, potential biases, generalisability, conflicts of interest and ethical considerations (Appendices B and C). A double‐rating approach was employed: the first reviewer (W.T.) rated all papers, while the second (M.T.) and third (D.C.) reviewers independently rated half. This cross‐checking ensured validation and minimised errors or biases. The risk of bias was classified as low, unclear or high for each study. Studies with appropriately recruited cohorts were rated as low risk, whereas those with unclear or unsuitable recruitment methods were rated as high or uncertain risk (Mathie et al. 2017).

### 2.3 Strategy for Data Analysis

Owing to significant data heterogeneity, a meta‐analysis was deemed unsuitable. Consequently, narrative synthesis was used to incorporate diverse research methodologies and achieve a comprehensive understanding. This approach involved summarising and interpreting study results through descriptive text, adhering to Popay et al. (2006) guidelines for structured data analysis. These guidelines begin with the development of a theory regarding how, why, and for whom the intervention works. A preliminary analysis was then conducted using data tabulation, which is displayed across various columns in a data extraction table (Table 1). Relationships within and between the studies were explored to deepen the analysis. Finally, the robustness of the synthesis was assessed and discussed to highlight the methodological and conceptual rigour of the included studies.

## 3 Results

A total of 27 studies were included in the review. Of these, 21 had quantitative designs (Costigan et al. 1997; Lustig and Thomas 1997; Ben‐Zur et al. 2005; Azar and Badr 2006; Van Riper 2007; Dukmak 2009; Gerstein et al. 2009; Lloyd and Hastings 2009; Hsiao and Van Riper 2011; Bayrakli and Kaner 2012; Truitt et al. 2012; Ellingsen et al. 2014; Hsiao 2014; Raspa et al. 2014; Choi and Yoo 2015; Rajan et al. 2016; Rajan and John 2017; Caples et al. 2018; Cless et al. 2018; Ginevra et al. 2018; Rajan et al. 2018) and six were qualitative (Grant and Whittell 2000; Gardner and Harmon 2002; Poehlmann et al. 2005; John and Zapata Roblyer 2017; Mohan and Kulkarni 2018; Karaca and Konuk 2021). There has been a growing interest in resilience among parents of children with intellectual disabilities over the past two decades (2000–2021). This is evident from the publication of 25 studies during this period, in contrast to only two studies published in 1997. To perform the preliminary synthesis, the data were tabulated in various columns of the same extraction table (Table 1).

### 3.1 Study Characteristics

#### 3.1.1 Countries of Study

The resulting search found relevant studies from 12 countries, Lebanon, *n* = 1 (Azar and Badr 2006); Turkey, *n* = 2 (Bayrakli and Kaner 2012; Karaca and Konuk 2021); Ireland, *n* = 1 (Caples et al. 2018); Israel, *n* = 1 (Ben‐Zur et al. 2005); USA, *n* = 9 (Costigan et al. 1997; Lustig and Thomas 1997; Poehlmann et al. 2005; Van Riper 2007; Gerstein et al. 2009; Truitt et al. 2012; Ellingsen et al. 2014; Raspa et al. 2014; Cless et al. 2018); Korea, *n* = 1 (Choi and Yoo 2015); UAE, *n* = 1 (Dukmak 2009); Australia, *n* = 1 (Gardner and Harmon 2002); Italy, *n* = 1 (Ginevra et al. 2018); Taiwan, *n* = 2 (Hsiao and Van Riper 2011; Hsiao 2014); India, *n* = 5 (Rajan et al. 2016, 2018; John and Zapata Roblyer 2017; Rajan and John 2017; Mohan and Kulkarni 2018); and the UK, *n* = 2 (Grant and Whittell 2000; Lloyd and Hastings 2009). The studies were conducted predominantly in Western (*n* = 13), Asian (*n* = 8) and Middle Eastern countries (*n* = 5).

#### 3.1.2 Study Samples

##### 3.1.2.1 Parents Characteristics

Of these 27 studies, 14 (51.9%) included parents (mothers and fathers) (Costigan et al. 1997; Lustig and Thomas 1997; Dukmak 2009; Gerstein et al. 2009; Lloyd and Hastings 2009; Hsiao and Van Riper 2011; Hsiao 2014; Choi and Yoo 2015; Rajan et al. 2016; Rajan and John 2017; Caples et al. 2018; Ginevra et al. 2018; Mohan and Kulkarni 2018; Rajan et al. 2018); 10 (37.0%) studies included only mothers (Gardner and Harmon 2002; Ben‐Zur et al. 2005; Poehlmann et al. 2005; Azar and Badr 2006; Van Riper 2007; Bayrakli and Kaner 2012; Ellingsen et al. 2014; John and Zapata Roblyer 2017; Cless et al. 2018; Karaca and Konuk 2021) and 3 (11.1%) studies included parents (mothers and fathers) and other family members (e.g., grandparents, sister, sibling) (Grant and Whittell 2000; Truitt et al. 2012; Raspa et al. 2014). Across the studies, the total sample size was 4433 participants, the majority of whom were mothers (*n* = 3592; 81.0%). Fathers represented a smaller proportion of individuals (*n* = 699; 15.8%). Two studies (Grant and Whittell 2000; Gerstein et al. 2009) did not separately report the sample numbers of mothers and fathers in their study.

##### 3.1.2.2 Children's Characteristics

All studies (*n* = 27, 100%) reported the age range of children with intellectual disabilities. Among these studies, the majority (*n* = 11, 40.74%) included children and adolescents ranging from 0 to 18 years old (Costigan et al. 1997; Gardner and Harmon 2002; Azar and Badr 2006; Van Riper 2007; Dukmak 2009; Lloyd and Hastings 2009; Hsiao and Van Riper 2011; Hsiao 2014; Choi and Yoo 2015; Cless et al. 2018; Karaca and Konuk 2021). Eight studies (29.63%) included children and younger adults ranging from 0 to 30 years old (Lustig and Thomas 1997; Poehlmann et al. 2005; Bayrakli and Kaner 2012; Rajan and John 2017; Caples et al. 2018; Ginevra et al. 2018; Mohan and Kulkarni 2018; Rajan et al. 2018). Five studies (18.52%) focused on children and adults aged 0–65 years (Grant and Whittell 2000; Ben‐Zur et al. 2005; Truitt et al. 2012; Raspa et al. 2014; Rajan et al. 2016), and three studies (11.11%) focused only on preschool children aged 3–6 years (Gerstein et al. 2009; Ellingsen et al. 2014; John and Zapata Roblyer 2017).

This systematic review aimed to explore and compare the resilience of parents caring for their children at different stages of the caregiving trajectory, specifically during childhood, adolescence, and adulthood. However, the studies included in this review often presented aggregated data that spanned a broad range of child ages from birth through adolescence to adulthood.

#### 3.1.3 Guiding Framework or Theory

The results reveal a diverse landscape in the utilisation of frameworks among studies. Only 12 studies identified their guiding framework; notably, four studies used the Resiliency model of Family Stress, Adjustment and Adaptation (Lustig and Thomas 1997; Van Riper 2007; Hsiao and Van Riper 2011; Caples et al. 2018). The remaining eight studies used different frameworks: Hope theory (Lloyd and Hastings 2009), the Model of Stress and Coping (Truitt et al. 2012), Olsson's model of resilience (Rajan et al. 2018), the Family Adjustment and Adaptation Response (FAAR) model (Choi and Yoo 2015; John and Zapata Roblyer 2017), Boss's Model of Family Stress (Cless et al. 2018), the double ABCX model of adjustment and adaptation (Costigan et al. 1997) and the ABCX model (Ellingsen et al. 2014).

### 3.2 Factors of Resilience

It is important to address the concern of comparability when different outcome measures of resilience are used across studies. Notably, using diverse outcome indicators does not inherently reduce comparability when resilience is conceptualised as a dynamic adaptive process. As noted above, resilience is defined as a process of positive adaptation in the context of significant adversity (Luthar and Cicchetti 2000). This means that various domains of well‐being can reflect that adaptation. For example, one study might assess resilience using parental mental health as an outcome, while another might use family functioning. Despite the different outcomes, both can signify that parents are managing to adapt successfully to their challenging circumstances. In a resilience framework, these different measures capture a facet of the same underlying phenomenon (successful adjustment and functioning despite adversity). Thus, outcomes such as strong mental health and high family functioning can be seen as valid indicators of resilience, grounded in the notion that the parent is doing well in the face of hardship. By recognising these outcomes as expressions of the resilience process, we maintain a common conceptual thread across studies (Walsh 2015). This approach preserves the meaningful comparability of findings, even if the specific outcome measures vary, because all are tied to the core definition of resilience as successful adaptation to stressors. In short, the heterogeneity in outcome measures reflects the multifaceted nature of resilience, rather than a limitation of the concept itself, allowing us to interpret diverse findings within one coherent resilience paradigm.

The categorization of factors into personal or contextual factors emerged directly from our analysis of the studies included in this systematic review. We systematically reviewed the literature to identify and synthesise factors that have been empirically linked to resilience across various populations and contexts (Table 2). During this process, we observed recurring themes and patterns that allowed us to categorise the factors into two main factors: personal factors and contextual factors. We used criteria similar to those outlined by Michalos (2014) to distinguish two main factors: personal factors related to an individual's values, beliefs and feelings (e.g., perception), health and body functions (e.g., physical health) and skills (e.g., problem‐solving, self‐efficacy), whereas contextual factors reflected social and environmental circumstances (e.g., social support, socioeconomic conditions, education, cultural or religious context).

**TABLE 2:** Summary of included studies investigating the factors associated with resilience.

	Author (year), Country	Stress	Problem‐solving	Perception of child's disability	Self‐efficacy	Hope	Parental health	Social support	Child's development	Child's age	Child behaviour	Socioeconomic situation	Education	Cultural and religious beliefs
1	Azar and Badr (2006)	✓	✓									✓	✓	
2	Bayrakli and Kaner (2012)		✓					✓						
3	Ben‐Zur et al. (2005)	✓			✓			✓				✓	✓	
4	Caples et al. (2018)		✓				✓	✓						
5	Choi and Yoo (2015)		✓				✓	✓	✓	✓				
6	Cless et al. (2018)		✓			✓								✓
7	Costigan et al. (1997)		✓											
8	Dukmak (2009)		✓											
9	Ellingsen et al. (2014)						✓		✓			✓	✓	
10	Gardner and Harmon (2002)		✓	✓	✓		✓	✓						✓
11	Gerstein et al. (2009)	✓												
12	Ginevra et al. (2018)				✓									
13	Grant and Whittell (2000)		✓											
14	Hsiao and Van Riper (2011)	✓		✓						✓				
15	Hsiao (2014)	✓						✓		✓		✓	✓	
16	John and Zapata Roblyer (2017)	✓		✓	✓			✓	✓		✓	✓		✓
17	Karaca and Konuk (2021)		✓	✓		✓								✓
18	Lloyd and Hastings (2009)					✓					✓			
19	Lustig and Thomas (1997), USA							✓						
20	Mohan and Kulkarni (2018)			✓	✓			✓	✓	✓	✓	✓	✓	
21	Poehlmann et al. (2005)		✓	✓				✓						✓
22	Rajan et al. (2016)												✓	
23	Rajan and John (2017)			✓					✓					
24	Rajan et al. (2018)		✓		✓									
25	Raspa et al. (2014)							✓				✓	✓	
26	Truitt et al. (2012)					✓								
27	Van Riper (2007)		✓				✓	✓						
	Total	6	13	7	6	4	4	12	5	4	3	7	7	5

#### 3.2.1 Personal Factors

Twenty‐four of the 27 studies presented information on personal factors, including stress, problem‐solving, perception, self‐efficacy, hope and parental health.

Family stress is a strong predictor of overall family functioning, particularly resilience (Hsiao 2014). Six studies examined the complex relationship between stress and resilience within families (Ben‐Zur et al. 2005; Azar and Badr 2006; Gerstein et al. 2009; Hsiao and Van Riper 2011; Hsiao 2014; John and Zapata Roblyer 2017). In the study by Azar and Badr (2006), parental stress, particularly among mothers, was found to be a significant predictor of maternal psychological well‐being and parental perceptions of their ability to cope with the challenges of raising a child with intellectual disabilities. Higher levels of parenting stress were strongly associated with poorer mental health outcomes in mothers, including increased depression and anxiety symptoms. Similarly, Ben‐Zur et al. (2005) showed that stress negatively correlates with mental health, social support, and hardiness, highlighting its detrimental effects on resilience‐related factors. Gerstein et al. (2009) and Hsiao and Van Riper (2011) extended this understanding by identifying mediating factors, such as family appraisal and positive parent–child relationships, which buffer stress impact and promote resilience. Hsiao (2014) highlighted that families with fewer stressors or better stress management exhibited higher resilience, crucial for maintaining family cohesion and functionality. In contrast, John and Zapata Roblyer (2017) used qualitative methods to demonstrate the tangible effects of financial and time‐related stressors on families. Their findings illustrate how such pressures can erode resilience, as evidenced by outcomes such as job losses and ongoing financial strains. In this context, job loss and ongoing financial strain are not operationalisations of resilience but rather indicators of its decline, showing how families struggle to adapt and recover when overwhelmed by stressors.

Problem‐solving is a cognitive process of identifying, analysing and implementing solutions to overcome obstacles or achieve goals (Weber 2005). Problem‐solving was reported in 13 studies (Costigan et al. 1997; Grant and Whittell 2000; Gardner and Harmon 2002; Poehlmann et al. 2005; Azar and Badr 2006; Van Riper 2007; Dukmak 2009; Bayrakli and Kaner 2012; Choi and Yoo 2015; Caples et al. 2018; Cless et al. 2018; Rajan et al. 2018; Karaca and Konuk 2021). A central theme across multiple studies has been the pivotal role of problem‐focused coping in enhancing resilience. Bayrakli and Kaner (2012) and Rajan et al. (2018) found that problem‐focused coping strategies significantly enhance resilience. Bayrakli and Kaner demonstrated a strong positive effect of problem‐focused coping on resilience, whereas Rajan et al. found that individuals with an internal locus of control who engaged in problem‐focused coping exhibited greater resilience. These findings are further supported by Van Riper (2007), who linked problem‐solving communication to higher levels of family adaptation. Caples et al. (2018) and Choi and Yoo (2015) emphasised the importance of communication skills in family functioning and adaptation. Caples et al. identified affirmative communication as a positive predictor of family functioning, whereas Choi and Yoo found a strong positive relationship between communication skills and family adaptation. This suggests that enhancing communication skills within families can improve their ability to adapt to stressors. Regarding gender and life stage, Costigan et al. (1997) found that mothers engage more in supportive problem‐solving and active listening than fathers, highlighting gender differences in coping that impact family resilience. Grant and Whittell (2000) noted that problem‐solving confidence varies by life stage, with parents of preschool children being less confident than older caregivers who show greater acceptance. These insights suggest that interventions aimed at boosting resilience should consider gender‐ and age‐specific strategies to address the unique challenges faced by different demographic groups.

Perception of a child's disability is another personal factor associated with resilience. Seven studies investigated parental perceptions (Gardner and Harmon 2002; Poehlmann et al. 2005; Gerstein et al. 2009; Hsiao and Van Riper 2011; John and Zapata Roblyer 2017; Rajan and John 2017; Mohan and Kulkarni 2018; Karaca and Konuk 2021). Gardner and Harmon (2002) provided insights into how resilient mothers maintain a positive outlook when managing challenges. Despite acknowledging the significant impact of having a child with a disability, these mothers did not feel that their life opportunities were diminished. Instead, they focused on enjoying life and utilising their abilities while caring for their families. Hsiao and Van Riper (2011) quantified the impact of family appraisals on family functioning, reporting a statistically significant correlation. This study showed that positive appraisal significantly enhances family functioning, which may foster resilience. Similarly, John and Zapata Roblyer (2017) highlighted the role of positive appraisals in resilience, as illustrated by a mother's admiration of her child's strength in overcoming early struggles. This aligns with the broader resilience literature, which emphasises the power of positive interpretations of difficult circumstances.

Self‐efficacy is another factor associated with resilience. Six studies investigated self‐efficacy using designs ranging from qualitative interviews (Gardner and Harmon 2002; Mohan and Kulkarni 2018) to quantitative surveys (Ben‐Zur et al. 2005; John and Zapata Roblyer 2017; Ginevra et al. 2018; Rajan et al. 2018). This diversity reflects the complexity of resilience and self‐efficacy, necessitating both subjective narratives and objective measures to capture these factors. Across these studies, a consistent finding was the role of self‐efficacy in fostering resilience. Ben‐Zur et al. (2005) emphasised that hardiness, related to self‐efficacy, is vital in assessing one's purpose and value, supporting resilience. Similarly, Ginevra et al. (2018) highlighted how individual resources such as control, curiosity, and confidence, which are components of self‐efficacy, contribute to resilience. This was echoed by Rajan et al. (2018), who found that parents with a strong internal locus of control, a concept similar to self‐efficacy, exhibited greater resilience. In qualitative insights, Gardner and Harmon (2002) focused on the influence of a stable childhood environment on developing a healthy sense of self, suggesting that positive early life experiences are foundational for later resilience and self‐efficacy. Mohan and Kulkarni (2018) explored how parents' past experiences enhance their self‐efficacy and resilience, suggesting that self‐efficacy is not only a result of current coping mechanisms but also a cumulative outcome of life experience.

Hope is the belief in a positive outcome, the ability to envision a better future, and the motivation to work towards that future (Snyder 2002). Four of the 24 included studies presented information on hope (Lloyd and Hastings 2009; Truitt et al. 2012; Cless et al. 2018; Karaca and Konuk 2021). Cless et al. (2018) and Truitt et al. (2012) emphasised the positive association between hope and caregiver adaptation. Cless et al. highlighted how hope helps mothers of children with Down syndrome manage stress, while Truitt et al. broadened this by showing that higher levels of hope correlate with better overall adaptation among caregivers. Lloyd and Hastings (2009) built on this understanding by examining how hope, as a psychological factor, influences mental health outcomes. This study did not measure resilience as an independent outcome but rather investigated hope's influence on mental health. By improving mental health, hope serves as a critical component of resilience, rather than a direct measure. This study is linked to Cless et al. (2018), who showed that hope is critical in reducing negative mental health outcomes such as stress, depression and anxiety in parents. Karaca and Konuk (2021) complement these studies by qualitatively exploring hope content. While other studies quantify hope's impact, Karaca and Konuk (2021) delve into what parents specifically hope for concerning their children's future, such as independence and self‐expression.

Parental health is closely tied to overall well‐being and can significantly impact a person's ability to cope with stress and adversity. Four studies reported that parents with good health are more likely to have better mental health and manage the emotional demands of raising a child with intellectual disabilities (Gardner and Harmon 2002; Van Riper 2007; Ellingsen et al. 2014; Choi and Yoo 2015). Choi and Yoo (2015) and Van Riper (2007) emphasised the importance of family resources, such as overall health, in promoting resilience. Choi and Yoo highlighted how parental health strengthens family units. Ellingsen et al. (2014) demonstrated the protective role of parental health in child development, particularly at age five. This study aligns with Choi and Yoo's emphasis on parental health but focuses on its direct impact on young children, suggesting that the benefits extend to the next generation. Additionally, Gardner and Harmon (2002) reported that mothers who care for their own well‐being are better equipped to support their children and other family members, linking parental self‐care to increased family resilience.

#### 3.2.2 Contextual Factors

Twenty of the 27 studies presented information on contextual factors, including social support, child characteristics, socioeconomic status, education and cultural and religious beliefs.

Social support is crucial for resilience. Parents with strong support networks, including family, friends and professionals, are more likely to be resilient. Twelve of the 20 studies discussed social support (Lustig and Thomas 1997; Gardner and Harmon 2002; Ben‐Zur et al. 2005; Poehlmann et al. 2005; Van Riper 2007; Bayrakli and Kaner 2012; Hsiao 2014; Raspa et al. 2014; Choi and Yoo 2015; John and Zapata Roblyer 2017; Caples et al. 2018; Mohan and Kulkarni 2018). Several studies explored social support's role in coping mechanisms and family adaptation. Bayrakli and Kaner (2012) indicated that higher social support levels positively associate with problem‐focused coping and resilience. Similarly, Ben‐Zur et al. (2005) found positive correlations between mental health scores and social support, indicating the critical role of social support in mental well‐being and resilience. Caples et al. (2018) and Lustig and Thomas (1997) examined social support within family systems, focusing on family functioning and adaptation as resilience indicators. Caples et al. (2018) demonstrated the importance of family communication, which supports resilience by strengthening family functioning and adaptive capacity. This suggests that resilience within families can be bolstered through strong social networks and communication. Similarly, Choi and Yoo (2015) found that family adaptation in Korean families is positively associated with family cohesiveness, supportive friends and available community services, showing that various sources of social support contribute to family resilience. Studies also highlighted the importance of professional support alongside informal networks (Gardner and Harmon 2002; Choi and Yoo 2015; John and Zapata Roblyer 2017; Mohan and Kulkarni 2018), providing specialised knowledge, therapeutic interventions and coping strategies.

Characteristics of a child as a contextual factor refers to an external element that can influence the demands placed on the parents, which can be grouped based on key child‐related factors: development (Ellingsen et al. 2014; Choi and Yoo 2015; John and Zapata Roblyer 2017; Rajan and John 2017; Mohan and Kulkarni 2018), age (Hsiao and Van Riper 2011; Hsiao 2014; Choi and Yoo 2015; Mohan and Kulkarni 2018), and behaviour (Lloyd and Hastings 2009; John and Zapata Roblyer 2017; Mohan and Kulkarni 2018). Rajan and John (2017) found that parents of children with severe intellectual disabilities experienced higher stress than those whose children had mild intellectual disabilities. Choi and Yoo (2015) reported that a child's developmental delays negatively impact family adaptation, causing stress to the parents. Ellingsen et al. (2014) found that a child's developmental difficulties negatively affect positive parenting, which is a critical aspect of parenting resilience. These studies suggest that developmental challenges can strain family relationships and reduce the effectiveness of positive parenting strategies. Regarding age, Hsiao and Van Riper (2011) and Hsiao (2014) identified that older children with Down syndrome are associated with healthier family functioning. This contrasts with the findings of Mohan and Kulkarni (2018), who observed that parents of younger children often reported higher stress due to their child's dependency, while parents of older children faced different challenges, such as behavioural problems and concerns about the future. These studies highlight how a child's age can shape the challenges a family faces, with younger children's dependency and older children's developmental milestones influencing family resilience. Mohan and Kulkarni (2018) also noted that parents of children with cooperative behaviour and fewer tantrums had more positive child‐rearing experiences. John and Zapata Roblyer (2017) further supported this by showing that a child's behaviour and developmental outcomes directly affect the family's ability to adapt to stressors.

Socioeconomic status (SES) was investigated in seven studies (Ben‐Zur et al. 2005; Azar and Badr 2006; Ellingsen et al. 2014; Hsiao 2014; Raspa et al. 2014; John and Zapata Roblyer 2017; Mohan and Kulkarni 2018). The impact of SES on resilience has been explored in diverse populations, outcomes and study designs, revealing both common themes and differences. Azar and Badr (2006) found that higher‐income families with medical insurance experience less strain than low‐income families without insurance. This suggests that financial stability and healthcare access are crucial for reducing stress and enhancing resilience. Hsiao (2014) found that higher family income correlates with healthier family functioning, suggesting that economic stability supports better family dynamics, promoting resilience. Conversely, Ellingsen et al. (2014) reported that lower income negatively affects positive parenting, which is a crucial component of resilience. This finding aligns with the studies by John and Zapata Roblyer (2017) and Raspa et al. (2014) further highlight the negative impact of financial strain on families. John and Zapata Roblyer (2017) noted that financial burdens of caring for children with intellectual disabilities often forced parents to leave full‐time employment, leading to significant financial stress and impacting the family's overall resilience.

Education of the parents and resilience‐related outcomes have been extensively studied across various contexts. Seven studies investigated education, with findings consistent with the significant role of education (Ben‐Zur et al. 2005; Azar and Badr 2006; Ellingsen et al. 2014; Hsiao 2014; Raspa et al. 2014; Rajan et al. 2016; Mohan and Kulkarni 2018). Azar and Badr (2006) reported that mothers with less education tend to have fewer coping resources, emphasising that lower educational attainment can limit resilience. Similarly, Ben‐Zur et al. (2005) found that mothers' years of education were positively correlated with mental health, social support and hardiness and negatively related to stress, suggesting that higher education levels enhance resilience by bolstering mental and social resources. In contrast, Mohan and Kulkarni (2018) found that lower socioeconomic status and higher parental education levels were associated with greater distress and less acceptance of a child's condition. This pattern varied across socioeconomic strata, indicating that the benefits of education on resilience may be context‐dependent and influenced by socioeconomic and cultural factors.

Cultural and religious beliefs were evident in five studies (Gardner and Harmon 2002; Poehlmann et al. 2005; John and Zapata Roblyer 2017; Cless et al. 2018; Karaca and Konuk 2021), shaping coping mechanisms and outcomes in challenging situations. Cless et al. (2018) found that greater religious coping was associated with increased levels of hope, supporting caregiver adaptation and indicating a link between religious engagement and positive psychological outcomes. Gardner and Harmon (2002) explored religious beliefs in an Australian context, where participants' beliefs ranged from reliance on God to secular faith in humanity, demonstrating that resilience can be supported by diverse beliefs depending on the cultural context. John and Zapata Roblyer (2017) examined how parents of children with intellectual disabilities framed caregiving within religious beliefs, viewing their child's disability as part of God's plan or influenced by Hindu Karma, providing purpose and strengthening resilience. Similarly, Karaca and Konuk (2021) studied mothers in Turkey and found that many drew comfort from Islamic practices, viewing their children's disabilities as Allah's will and their caregiving role as a spiritual test leading to divine reward. This cultural context highlights how religious beliefs influence resilience by providing meaning and hope. Poehlmann et al. (2005) also reported that faith plays a crucial role in coping, with individuals often interpreting difficult circumstances as part of God's perfect timing, helping them accept and find peace in their situations.

#### 3.2.3 Synthesise of Finding: A Conceptual Model of Resilience for Parents of Children With Intellectual Disabilities (Justification for the Conceptual Model of Resilience)

**FIGURE 2:**
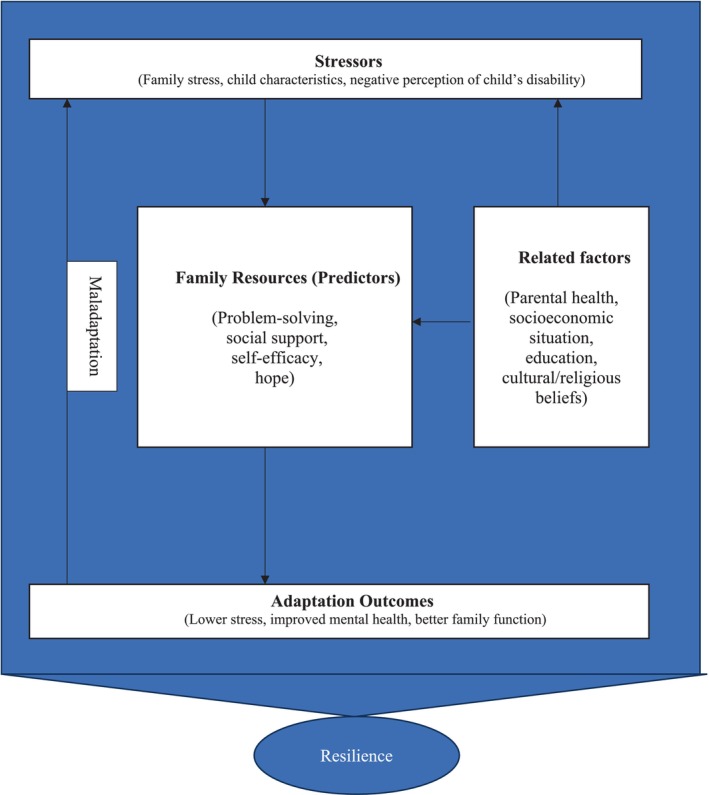
Conceptual model of resilience for parents of children with intellectual disabilities. The figure represents the Conceptual Model of Resilience for Parents of Children with Intellectual Disabilities, based on the Resiliency Model of Family Stress, Adjustment, and Adaptation (McCubbin et al. 1996).

This study employs the Resiliency Model of Family Stress, Adjustment, and Adaptation (McCubbin et al. 1996) as a guiding framework to synthesise and organise the factors associated with resilience in parents of children with intellectual disabilities. This theoretical model helps explain how families respond to and manage stressors by activating resources and coping strategies, leading to adaptation or maladaptation. The conceptual model developed (Figure 2) organises key variables into four primary domains: Stressors, Family Resources (Predictors), Related factors and Adaptation Outcomes, concluding to the construct of Resilience.

##### 3.2.3.1 Stressors

Stressors/challenges represent the initial demands placed on parents due to their child's intellectual disabilities and caregiving responsibilities. Key stressors include family stress, child characteristics and negative perceptions of the child's disability.

Family stress is a central tenet of the Resiliency Model, and the sources consistently highlight that raising a child with intellectual disabilities is a significant stressor for families (Azar and Badr 2006; Gerstein et al. 2009; Bayrakli and Kaner 2012). The “double ABCX model” emphasises the stress of having a child with special needs and how families cope with these demands (Azar and Badr 2006). Research indicates that parents of children with disabilities confront many difficulties caused by the disability, in addition to typical parental stress (Bayrakli and Kaner 2012). Studies have found higher stress levels in parents of children with intellectual disabilities than in parents of typically developing children (Gerstein et al. 2009). This stress can be related to various factors, including the child's diagnosis and behavioural problems, leading to a pile‐up of demands (John and Zapata Roblyer 2017).

Child characteristics of intellectual disabilities can act as stressors. For example, the developmental level of the child (Choi and Yoo 2015) and mobility/independence issues can contribute to parental stress (Mohan and Kulkarni 2018). Behaviour problems are frequently cited as significant stressors for parents of children with ID (Gerstein et al. 2009; Raspa et al. 2014). The initial diagnosis of a disability can be a stressor, challenging parents' expectations (Bayrakli and Kaner 2012; John and Zapata Roblyer 2017).

Negative perception of a child's disability has been found to be a strong predictor of parental stress (John and Zapata Roblyer 2017). Viewing the impact of the child's condition negatively can worsen parental well‐being (Caples et al. 2018). Conversely, more positive interpretations of rearing a child with a disability are associated with better adaptation (Hsiao and Van Riper 2011; Mohan and Kulkarni 2018).

Family resources (predictors) consists of critical predictors of resilience that empower parents to manage stress effectively, including problem‐solving, social support, self‐efficacy, and hope.

Problem‐solving skills are identified as a key component influencing a family's ability to adapt within the Resiliency Model (Hsiao and Van Riper 2011; Caples et al. 2018). Problem‐focused coping strategies are important protective factors for maternal resilience (Bayrakli and Kaner 2012). Supporting parents in using problem‐focused coping strategies is recommended to promote parental resilience (Bayrakli and Kaner 2012). Resilience in families caring for a child with intellectual disabilities is associated with the propensity to concentrate on difficult situations and search for possible solutions (problem‐focused coping) (Ginevra et al. 2018).

Social support is consistently identified as a vital resource influencing parents' coping strategies and resilience (Bayrakli and Kaner 2012; John and Zapata Roblyer 2017; Caples et al. 2018). It affects the cognitive appraisal of adversity and is a determinant of coping strategies (Bayrakli and Kaner 2012). Increased social support can enrich coping strategies and increase the hardiness of mothers, making them more resilient (Bayrakli and Kaner 2012). Research indicates that the availability of quality social support is a strong predictor of family adaptation (Caples et al. 2018). Families with strong informal support networks tend to have more positive outcomes (Raspa et al. 2014).

The term “self‐efficacy” is not as explicitly and frequently mentioned across all sources; the concept is related to a parent's perceived competence and ability to manage the demands of raising a child with intellectual disabilities. Lustig and Thomas (1997) found that families who perceived themselves as competent had better family adjustment (Mohan and Kulkarni 2018). The “Condition Management Ability Scale” assesses parents' perceptions of their competence in caring for their child's condition (Hsiao and Van Riper 2011). A sense of coherence, which includes viewing a crisis as manageable, is negatively related to parents' perceived stress (John and Zapata Roblyer 2017).

Hope is a significant factor in adapting to the uncertainty associated with caring for a child with Down syndrome (Truitt et al. 2012; Cless et al. 2018). Caregivers' level of hope has been found to be a significant predictor of overall adaptation (Truitt et al. 2012). Hope may contribute to mothers' overall functioning and is considered a part of the perception component in the contextual model of family stress (Cless et al. 2018). Resilience in families caring for a child with intellectual disabilities can also be linked to the tendency to look for and expect positive outcomes when confronting problems (optimism, which is closely related to hope) (Ginevra et al. 2018).

Related factors are generally context‐shaping rather than core resources or stressors. However, depending on the circumstances, they may operate as either. They influence the family's access to resources, appraisal of the situation, and overall capacity for resilience, including parental health, socioeconomic situation, education and cultural/religious beliefs.

Parental physical health can significantly affect a parent's ability to cope with the demands of raising a child with intellectual disabilities (Ellingsen et al. 2014; Choi and Yoo 2015; John and Zapata Roblyer 2017). For example, parents' health is positively related to family adaptation (Choi and Yoo 2015).

Socioeconomic situations, including financial resources and socioeconomic backgrounds, can act as moderators of stress and influence the availability of resources and coping strategies (Ellingsen et al. 2014; John and Zapata Roblyer 2017; Mohan and Kulkarni 2018). Financial strain is associated with elevated stress levels in families (John and Zapata Roblyer 2017).

Education, particularly the mother's education level, has been suggested as a protective factor for positive parenting in the face of risk (Ellingsen et al. 2014; Rajan et al. 2018). Mothers with more education may engage in more positive parenting at higher risk levels (Ellingsen et al. 2014).

Cultural and religious beliefs can influence parents' understanding of their child's disability and the coping mechanisms they employ (John and Zapata Roblyer 2017; Mohan and Kulkarni 2018). For instance, religious affiliation has been reported to alleviate stress and enhance coping (Mohan and Kulkarni 2018).

Adaptation outcomes taken together these perspectives frame resilience as an ongoing adaptive process rather than a static attribute. Within this framework, the ultimate proof of resilience lies in attaining positive adaptive outcomes, despite adverse conditions. The findings suggest that resilience manifests as lower stress, improved mental health and better family functioning.

Lower stress is a key indicator of positive adaptation. Resilience processes aim to minimise or decrease parents' stress responses over time (Gerstein et al. 2009; Cless et al. 2018; Rajan et al. 2018).

Improved mental health in parents is a significant outcome associated with resilience (Ben‐Zur et al. 2005; John and Zapata Roblyer 2017; Ginevra et al. 2018). Parents who cope better with stressful demands often experience higher levels of life satisfaction and a more positive mental health status (Ginevra et al. 2018).

Better family functioning signifies successful adaptation at the family level. This includes maintaining family routines, positive interactions, and the overall well‐being of the family unit despite challenges (Van Riper 2007; Hsiao and Van Riper 2011; Hsiao 2014; Caples et al. 2018). This can manifest as effective problem‐solving, communication and emotional responsiveness within the family (Hsiao and Van Riper 2011).

By synthesising theoretical frameworks and findings, the model offers a comprehensive lens for understanding resilience in parents of children with intellectual disabilities (Figure 2).

## 4 Discussion

This systematic review investigated the factors associated with resilience in parents of children with intellectual disabilities. Understanding these factors is important, as parenting a child with an intellectual disability has been reported to impact the mental health and well‐being of family caregivers (Rydzewska et al. 2021). However, there may be a gender bias in the findings, since mothers are overrepresented and fathers are underrepresented in this review. Research has indicated that biases in recruitment processes can result in the underrepresentation or overrepresentation of specific genders, thereby affecting the generalisability of research findings (Moss‐Racusin et al. 2012; Xiao et al. 2018). It is crucial to provide more consideration to this group of parents and support them in coping with and adapting to the challenges they face. This review provides valuable insights into the factors associated with resilience in parents of children with intellectual disabilities and highlights the need for better support and services for these parents to improve their resilience. According to Masten and Reed (2002), research on resilience began in the 1960s. However, the first reported studies on resilience in parents of children with intellectual disabilities were only reported in 1997 (Costigan et al. 1997; Lustig and Thomas 1997).

This finding is striking since parents of children with intellectual disabilities are at a higher risk of mental health problems such as anxiety, depression and stress (Shin and Crittenden 2003; McConkey et al. 2008; Dunn et al. 2019). However, resilience may buffer these adverse impacts. From this review, the models which best capture the findings on how stressors (e.g., child behaviour, financial strain) interact with resources (e.g., social support, problem‐solving) to drive adaptation at the individual and family levels include: the Resiliency Model of Family Stress Adjustment, and Adaptation (McCubbin et al. 1996), the Family Adjustment and Adaptation Response (FAAR) (Patterson 1988), which was developed from the Double ABCX Model of Adjustment and Adaptation (McCubbin and Patterson 1983) and the ABCX Model (Hill 1949). Boss's Model of Family Stress (Boss et al. 2016) addresses the distinction between external and internal contexts surrounding a stressor and should guide future studies in modelling the contextual moderators of adaptation. Olsson's Model of Resilience (Olsson 2008) is valuable for selecting risk and protective covariates, although it is less process‐oriented. Hope Theory (Snyder 2002) helps explain why beliefs and pathway thinking co‐vary with better adaptation, and the Model of Stress and Coping (Lazarus and Folkman 1984) highlights personal and environmental factors; however, these two theories are less explicit about family adaptation.

In terms of gender roles and societal expectations may influence how resilience is expressed and perceived by different caregivers. Traditionally, women have often been associated with nurturing and caregiving roles, which may lead to the assumption that mothers are more resilient (Sharma et al. 2016). However, resilience is not solely determined by gender or caregiving roles; it is also influenced by individual characteristics and circumstances. Studies have explored resilience among various caregivers, including mothers, fathers and other family members (Rajan and John 2017; Caples et al. 2018; Mohan and Kulkarni 2018). These studies have shown that individuals may exhibit resilience in different ways, influenced by factors such as social support, problem‐solving, hope and the specific challenges faced by each caregiver (Peer and Hillman 2014; Arzeen et al. 2020; Tejakum et al. 2022; Cheng et al. 2024). When examining resilience, it is important to consider the unique experiences and contexts of individual caregivers.

According to previous studies, there is a dynamic link between personal and contextual factors of resilience. Resilience is determined by both personal and contextual factors (Jaffee et al. 2007; Ungar 2008). Regarding contextual factors, Machalicek et al. (2015) emphasised the importance of education and training programs to improve parents' ability to effectively support children with intellectual disabilities. The factors identified in this systematic review highlight the need for professional support to help parents acquire realistic interventions that promote resilience. A “realistic intervention” refers to an approach that is feasible, evidence‐based and tailored to the unique needs of parents and children. Informal support from family, friends, support groups, and community resources can also play a crucial role. The emphasis on formal support may stem from healthcare professionals offering specialised knowledge, resources and structured guidance that are not always available through informal channels. Integrating formal and informal support can provide a more comprehensive system for parents.

Finally, it is important to note that resilience is a dynamic process that can vary over time and across situations, making parents resilient in one situation but not necessarily in another situation. Each stage presents unique challenges and stressors for parents, requiring different types of support to navigate effectively (Haveman et al. 1997; Heller et al. 1997; Chiu et al. 2013; Schulz et al. 2020). However, this systematic review lacks a detailed description of caregiving for children with intellectual disabilities across various phases. This absence of a phase‐wise breakdown limits our understanding of the unique challenges and stressors that parents face at each stage of caregiving. It is challenging to discern whether certain interventions or factors are more effective at specific points in the caregiving journey. Future research should consider conducting analyses stratified by caregiving phase to enhance the applicability of the findings and offer more targeted recommendations. This approach would contribute to a more nuanced understanding of caregivers' experiences at different points in their journey and assist in tailoring support interventions more effectively to parents' specific needs at each stage.

### 4.1 Strengths and Limitations

This study provides a foundational understanding for future research and for parents and professionals regarding resilience in parents of children with intellectual disabilities. By systematically reviewing key studies, it provides an overview of the current knowledge by employing rigorous methods to minimise bias and ensure validity and reliability. The synthesis of multiple studies enhances the robustness of the findings. However, this study has some limitations. Despite a comprehensive search strategy, it may not capture all research on the intervention factors associated with resilience. Future research should focus on intervention studies that examine various interventions and their impact on parental resilience. Additionally, most included studies showed an unclear risk of bias in 1–2 domains according to the CASP criteria, with an average score between B1 and B2 (CASP 2018). Quantitative studies often lack precision regarding confidence intervals, whereas qualitative studies frequently do not clarify the researcher–participant relationship. Finally, a meta‐analysis could not be conducted because of the differing resilience measures.

## 5 Conclusion

This systematic review identified several factors associated with resilience in parents of children with intellectual disabilities. These factors may not only contribute to resilience but could also be concomitant with or the outcomes of resilience. For instance, parents' ability to find meaning and purpose in raising a child with intellectual disabilities can be influenced by their resilience. Parental resilience is an ongoing process subject to various personal and contextual factors over time. Further research is needed to understand the complex interactions between these factors and develop interventions that effectively enhance resilience. Recognising these factors helps us understand the unique challenges parents face and develop supportive solutions. This can lead to improved health services, better outcomes for emotional disorders (e.g., anxiety, stress and depression), and aid parents in adapting to their challenges, thereby maintaining their overall resilience.

## Author Contributions

Mr. Wattana Tejakum conceptualised and designed the study, conducted the literature review, and wrote the manuscript. Dr. Maria Truesdale contributed to data analysis, interpretation of results and provided critical revisions. Prof. Craig Melville assisted in the methodology design and reviewed the study selection. Prof. Deborah Cairns contributed to the writing of the discussion and reviewed the manuscript for important content.

## Funding

This research was funded by the Royal Thai Government Scholarship, under grant PH_G5620.

## Ethics Statement

The authors have nothing to report.

## Conflicts of Interest

The authors declare no conflicts of interest.

## Data Availability

The data that support the findings of this study are available from the corresponding author, upon reasonable request.

## Appendix A Search Strategy

### Search Method

A systematic search was conducted using five databases: Medical Literature Analysis and Retrieval System Online (MEDLINE), Psychological Information Database (PsycINFO), Excerpta Medica dataBASE (EMBASE), Cumulative Index to Nursing and Allied Health Literature (CINAHL), and Sociology research database (SocINDEX).

### Electronic Search

We conducted electronic searches by utilising the Ovid EMBASE search approach and modifying it as needed for other databases

1. exp. parent/
2. parent*.mp. [mp = title, book title, abstract, original title, name of substance word, subject heading word, floating sub‐heading word, keyword heading word, organism supplementary concept word, protocol supplementary concept word, rare disease supplementary concept word, unique identifier, synonyms, population supplementary concept word, anatomy supplementary concept word]
3. mother*.mp. [mp = title, book title, abstract, original title, name of substance word, subject heading word, floating sub‐heading word, keyword heading word, organism supplementary concept word, protocol supplementary concept word, rare disease supplementary concept word, unique identifier, synonyms, population supplementary concept word, anatomy supplementary concept word]
4. father*.mp. [mp = title, book title, abstract, original title, name of substance word, subject heading word, floating sub‐heading word, keyword heading word, organism supplementary concept word, protocol supplementary concept word, rare disease supplementary concept word, unique identifier, synonyms, population supplementary concept word, anatomy supplementary concept word]
5. 1 or 2 or 3 or 4
6. exp psychological resilience/
7. resilien*.mp. [mp = title, book title, abstract, original title, name of substance word, subject heading word, floating sub‐heading word, keyword heading word, organism supplementary concept word, protocol supplementary concept word, rare disease supplementary concept word, unique identifier, synonyms, population supplementary concept word, anatomy supplementary concept word]
8. coping.mp. [mp = title, book title, abstract, original title, name of substance word, subject heading word, floating sub‐heading word, keyword heading word, organism supplementary concept word, protocol supplementary concept word, rare disease supplementary concept word, unique identifier, synonyms, population supplementary concept word, anatomy supplementary concept word]
9. cope.mp. [mp = title, book title, abstract, original title, name of substance word, subject heading word, floating sub‐heading word, keyword heading word, organism supplementary concept word, protocol supplementary concept word, rare disease supplementary concept word, unique identifier, synonyms, population supplementary concept word, anatomy supplementary concept word]
10. adapt*.mp. [mp = title, book title, abstract, original title, name of substance word, subject heading word, floating sub‐heading word, keyword heading word, organism supplementary concept word, protocol supplementary concept word, rare disease supplementary concept word, unique identifier, synonyms, population supplementary concept word, anatomy supplementary concept word]
11. hardiness.mp. [mp = title, book title, abstract, original title, name of substance word, subject heading word, floating sub‐heading word, keyword heading word, organism supplementary concept word, protocol supplementary concept word, rare disease supplementary concept word, unique identifier, synonyms, population supplementary concept word, anatomy supplementary concept word]
12. 6 or 7 or 8 or 9 or 10 or 11
13. 5 and 12
14. exp child/
15. child*.mp. [mp = title, book title, abstract, original title, name of substance word, subject heading word, floating sub‐heading word, keyword heading word, organism supplementary concept word, protocol supplementary concept word, rare disease supplementary concept word, unique identifier, synonyms, population supplementary concept word, anatomy supplementary concept word]
16. 14 or 15
17. exp intellectual impairment/
18. mental retardation.mp. [mp = title, book title, abstract, original title, name of substance word, subject heading word, floating sub‐heading word, keyword heading word, organism supplementary concept word, protocol supplementary concept word, rare disease supplementary concept word, unique identifier, synonyms, population supplementary concept word, anatomy supplementary concept word]
19. intellectual disabilit*.mp. [mp = title, book title, abstract, original title, name of substance word, subject heading word, floating sub‐heading word, keyword heading word, organism supplementary concept word, protocol supplementary concept word, rare disease supplementary concept word, unique identifier, synonyms, population supplementary concept word, anatomy supplementary concept word]
20. Down syndrome.mp. [mp = title, book title, abstract, original title, name of substance word, subject heading word, floating sub‐heading word, keyword heading word, organism supplementary concept word, protocol supplementary concept word, rare disease supplementary concept word, unique identifier, synonyms, population supplementary concept word, anatomy supplementary concept word]
21. learning disabilit*.mp. [mp = title, book title, abstract, original title, name of substance word, subject heading word, floating sub‐heading word, keyword heading word, organism supplementary concept word, protocol supplementary concept word, rare disease supplementary concept word, unique identifier, synonyms, population supplementary concept word, anatomy supplementary concept word]
22. developmental disabilit*.mp. [mp = title, book title, abstract, original title, name of substance word, subject heading word, floating sub‐heading word, keyword heading word, organism supplementary concept word, protocol supplementary concept word, rare disease supplementary concept word, unique identifier, synonyms, population supplementary concept word, anatomy supplementary concept word]
23. 17 or 18 or 19 or 20 or 21 or 22
24. 16 and 23
25. 13 and 24
26. limit 25 to (human and English language)

### Hand‐Searching

We also reviewed the reference lists of the included studies, applying both backward and forward reference searching techniques to ensure that they did not miss any relevant publications.

## Appendix B Quality Assessment for Quantitative Studies

**Table:**

CASP checklist	Azar and Badr (2006)	Bayrakli and Kaner (2012)	Ben‐Zur et al. (2005)	Caples et al. (2018)	Choi and Yoo (2015)
1. Did the study address a clearly focused issue?	Yes	Yes	Yes	Yes	Yes
2. Was the cohort recruited in an acceptable way?	Yes	Yes	Yes	Yes	Yes
3. Was the exposure accurately measured to minimise bias?	Yes	Yes	Yes	Yes	Yes
4. Was the outcome accurately measured to minimise bias?	Yes	Yes	Yes	Yes	Yes
5. (a) Have the authors identified all important confounding factors?	Yes	Yes	Yes	Yes	Yes
5. (b) Have they taken account of the confounding factors in the design and/or analysis?	Yes	Yes	Yes	Yes	Yes
6. (a) Was the follow up of subjects complete enough?	Yes	Yes	Yes	Yes	Yes
6. (b) Was the follow up of subjects long enough?	Yes	Yes	Yes	Yes	Yes
7. What are the results of this study?	Yes	Yes	Yes	Yes	Yes
8. How precise are the results?	Can't Tell	Yes	Can't Tell	Can't Tell	Can't Tell
9. Do you believe the results?	Yes	Yes	Yes	Yes	Yes
10. Can the results be applied to the local population?	Yes	Yes	Yes	Yes	Can't Tell
11. Do the results of this study fit with other available evidence?	Yes	Yes	Yes	Yes	Yes
12. What are the implications of this study for practice?	Yes	Yes	Yes	Yes	Yes
Total score	B1	A	B1	B1	B2

CASP checklist	Cless et al. (2018)	Costigan et al. (1997)	Dukmak (2009)	Ellingsen et al. (2014)	Gerstein et al. (2009)
1. Did the study address a clearly focused issue?	Yes	Yes	Yes	Yes	Yes
2. Was the cohort recruited in an acceptable way?	Yes	Yes	Yes	Yes	Yes
3. Was the exposure accurately measured to minimise bias?	Can't Tell	Yes	Yes	Yes	Yes
4. Was the outcome accurately measured to minimise bias?	Yes	No	Yes	Yes	Yes
5. (a) Have the authors identified all important confounding factors?	Yes	Can't Tell	Yes	Yes	Yes
5. (b) Have they taken account of the confounding factors in the design and/or analysis?	Yes	Can't Tell	Yes	Yes	Yes
6. (a) Was the follow up of subjects complete enough?	Yes	Yes	Yes	Yes	Yes
6. (b) Was the follow up of subjects long enough?	Yes	Yes	Yes	Yes	Yes
7. What are the results of this study?	Yes	Yes	Yes	Yes	Yes
8. How precise are the results?	Yes	Can't Tell	Can't Tell	Yes	Can't Tell
9. Do you believe the results?	Yes	Yes	Yes	Yes	Yes
10. Can the results be applied to the local population?	Yes	Yes	Can't Tell	Yes	Yes
11. Do the results of this study fit with other available evidence?	Yes	Yes	Yes	Yes	Yes
12. What are the implications of this study for practice?	Yes	Can't Tell	Yes	Yes	Can't Tell
Total score	B1	C1,4	B2	A	B2

CASP checklist	Ginevra et al. (2018)	Hsiao and Van Riper (2011)	Hsiao (2014)	Lloyd and Hastings (2009)	Lustig and Thomas (1997)	Rajan et al. (2016)
1. Did the study address a clearly focused issue?	Yes	Yes	Yes	Yes	Yes	Yes
2. Was the cohort recruited in an acceptable way?	Yes	Yes	Yes	Yes	Yes	Yes
3. Was the exposure accurately measured to minimise bias?	Yes	Yes	Yes	Yes	Yes	Yes
4. Was the outcome accurately measured to minimise bias?	Yes	Yes	Yes	Yes	Yes	Yes
5. (a) Have the authors identified all important confounding factors?	Yes	Yes	Yes	Yes	Yes	Yes
5. (b) Have they taken account of the confounding factors in the design and/or analysis?	Yes	Yes	Yes	Yes	Yes	Yes
6. (a) Was the follow up of subjects complete enough?	Yes	Yes	Yes	Yes	Yes	Yes
6. (b) Was the follow up of subjects long enough?	Yes	Yes	Yes	Yes	Yes	Yes
7. What are the results of this study?	Yes	Yes	Yes	Yes	Yes	Yes
8. How precise are the results?	Yes	Can't Tell	Can't Tell	Can't Tell	Yes	Can't Tell
9. Do you believe the results?	Yes	Yes	Yes	Yes	Yes	Yes
10. Can the results be applied to the local population?	Can't Tell	Can't Tell	Can't Tell	Can't Tell	Yes	Can't Tell
11. Do the results of this study fit with other available evidence?	Yes	Yes	Yes	Yes	Yes	Yes
12. What are the implications of this study for practice?	Yes	Yes	Yes	Yes	Can't Tell	Yes
Total score	B1	B2	B2	B2	B1	B2

CASP checklist	Rajan and John (2017)	Rajan et al. (2018)	Raspa et al. (2014)	Riper (2007)	Truitt et al. (2012)
1. Did the study address a clearly focused issue?	Yes	Yes	Yes	Yes	Yes
2. Was the cohort recruited in an acceptable way?	Yes	Yes	Yes	Yes	Yes
3. Was the exposure accurately measured to minimise bias?	Yes	Yes	Yes	Yes	Yes
4. Was the outcome accurately measured to minimise bias?	Yes	Yes	Yes	Yes	Yes
5. (a) Have the authors identified all important confounding factors?	Yes	Yes	Yes	Yes	Yes
5. (b) Have they taken account of the confounding factors in the design and/or analysis?	Yes	Yes	Yes	Yes	Yes
6. (a) Was the follow up of subjects complete enough?	Yes	Yes	Yes	Yes	Yes
6. (b) Was the follow up of subjects long enough?	Yes	Yes	Yes	Yes	Yes
7. What are the results of this study?	Yes	Yes	Yes	Yes	Yes
8. How precise are the results?	Can't Tell	Can't Tell	Yes	Can't Tell	Can't Tell
9. Do you believe the results?	Yes	Yes	Yes	Yes	Yes
10. Can the results be applied to the local population?	Yes	Can't Tell	Can't Tell	Can't Tell	Yes
11. Do the results of this study fit with other available evidence?	Yes	Yes	Yes	Yes	Yes
12. What are the implications of this study for practice?	Yes	Can't Tell	Can't Tell	Yes	Yes
Total score	B1	B3	B2	B2	B1

*Note:* Rating A = low risk of bias in all domains; Rating B*x* = unclear risk of bias in *x* domains, low risk of bias in all other domains; Rating C*xy* = high risk of bias in *y* domains, unclear risk of bias in *x* domains, low risk of bias in all other domains.

## Appendix C Quality Assessment for Qualitative Studies

**Table:**

CASP checklist	Gardner and Harmon (2002)	Grant and Whittell (2000)	John and Zapata Roblyer (2017)	Karaca and Sener (2021)	Mohan and Kulkarni (2018)	Poehlmann et al. (2005)
1. Was there a clear statement of the aims of the research?	Yes	Yes	Yes	Yes	Yes	Yes
2. Is a qualitative methodology appropriate?	Yes	Yes	Yes	Yes	Yes	Yes
3. Was the research design appropriate to address the aims of the research?	Yes	Yes	Yes	Yes	Yes	Yes
4. Was the recruitment strategy appropriate to the aims of the research?	Yes	Yes	Yes	Yes	Yes	Yes
5. Was the data collected in a way that addressed the research issue?	Yes	Yes	Yes	Yes	Yes	Yes
6. Has the relationship between researcher and participants been adequately considered?	Can't Tell	Yes	Can't Tell	Can't Tell	Can't Tell	Can't Tell
7. Have ethical issues been taken into consideration?	Can't Tell	Can't Tell	Yes	Yes	Can't Tell	Can't Tell
8. Was the data analysis sufficiently rigorous?	Can't Tell	Yes	Yes	Yes	Yes	Yes
9. Is there a clear statement of findings?	Yes	Yes	Yes	Yes	Yes	Yes
10. How valuable is the research?	Yes	Yes	Yes	Yes	Yes	Yes
Total score	B3	B1	B1	B1	B2	B2

*Note:* Rating A = low risk of bias in all domains; Rating B*x* = unclear risk of bias in *x* domains, low risk of bias in all other domains; Rating C*xy* = high risk of bias in *y* domains, unclear risk of bias in *x* domains, low risk of bias in all other domains.
